# Biocompatible nanoparticles self-assembled by PEGylated polyphosphoesters for combination of photodynamic therapy and hypoxia-activated chemotherapy against breast cancer

**DOI:** 10.3389/fphar.2024.1529631

**Published:** 2024-12-23

**Authors:** Fengyu Wang, Xiaojing Zou, Chunyang Sun

**Affiliations:** Department of Radiology, Tianjin Key Laboratory of Functional Imaging and Tianjin Institute of Radiology, Tianjin Medical University General Hospital, Tianjin, China

**Keywords:** polyphosphoesters, nanocarriers, photodynamic therapy, hypoxia-activated chemotherapy, combination therapy, cancer treatment

## Abstract

**Introduction:**

Although photodynamic therapy (PDT) shows considerable potential for cancer treatment due to its precise spatial control and reduced toxicity, effectively eliminating residual cells under hypoxic conditions remains challenging because of the resistance conferred by these cells.

**Methods:**

Herein, we synthesize an amphiphilic PEGylated polyphosphoester and present a nanocarrier (NP_CT_) specifically designed for the codelivery of hydrophobic photosensitizer (chlorin e6, Ce6) and hypoxia-activated prodrugs (tirapazamine, TPZ). We investigate the antitumor effect of NP_CT_ on both cellular and animal level.

**Results:**

The efficient encapsulation of Ce6 and TPZ by NP_CT_ enables the prolonged blood circulation and improved tumor distribution of both agents. Upon internalization by tumoral cells, 660 nm laser irradiation activates Ce6, leading to the generation of reactive oxygen species (ROS) that effectively kill murine 4T1 breast cancer cells. Meanwhile, the PDT process consumes a large amount of oxygen to generate the hypoxic microenvironment that activates the liberated TPZ from NP_CT_. The resulting highly cytotoxic radicals specifically target and induce cytotoxicity in remaining hypoxic cancer cells. Compared to other groups, the combination of NP_CT_ and 660 nm laser irradiation resulted in the most substantial tumor growth inhibition.

**Discussion:**

This innovative approach provides new avenues for the development of advanced delivery systems based on polyphosphoesters and combination therapeutic strategies.

## Introduction

Breast cancer has become one of the two most prevalent cancers worldwide, posing a serious threat to human health (Arnold et al., 2022; Lei et al., 2021; Qian et al., 2023). Photodynamic therapy (PDT) is an advanced therapeutic modality that utilizes a photosensitizing agent, light, and oxygen to produce cytotoxic reactive-oxygen-species (ROS) for selective cancer cells killing (Li X. et al., 2020; Ji et al., 2022; Lam et al., 2023). Compared to traditional radiotherapy, PDT could considerably reduce systemic toxicity due to the precise spatial controllability and biosafety of the red or near infrared red (NIR) light used (Yao et al., 2023; Zhou et al., 2023; Dolmans et al., 2003). Although the patients receiving PDT need precautions against sunlight exposure post-treatment, overall systemic side effects of PDT are obviously decreased (Zhang et al., 2024; Brown et al., 2004). More importantly, PDT has been found to be combined with chemotherapy to realize the synergistic therapy against malignant cancers (Lee et al., 2018; Jia et al., 2021; Canti et al., 1998). For instance, PDT can improve the permeability of cancer cells through ROS-disrupted cell membranes and altered cell structure, thereby increasing the uptake of chemotherapy agents (He et al., 2015). Recent studies also demonstrated that PDT can induce a transient arrest in the cell cycle, particularly in the G2/M phase, making the cancer cells more susceptible to specific chemotherapy (Moloudi et al., 2023; Hu et al., 2021).

Significant hypoxic conditions arise following conventional PDT due to the depletion of surrounding O_2_ level and alterations in associated physiological processes (Li et al., 2022; Pucelik et al., 2020). It is important to highlight that the hypoxia generated by PDT may enhance the resistance of cancer cells to treatment via multiple mechanisms (Muz et al., 2015; Li D. et al., 2023; Jing et al., 2019; Li W. et al., 2020; Wilson and Pay, 2011). For instance, cycles of hypoxia and subsequent reoxygenation during PDT can lead to the stimulation and stabilization of hypoxia-inducible factor-1α (HIF-1α), and HIF-1α independently enhances the expression of downstream genes, such as p53 and c-Myc, which play crucial roles in regulating cell death and survival (Greijier and Wall, 2004; Paredes et al., 2021; Qian et al., 2020). Conversely, a hypoxic environment prevents PDT from fully utilizing oxygen to generate ROS, resulting in a decrease in PDT efficacy (Chen et al., 2021; Li J. et al., 2023). As a result, the residual cancer cells in hypoxic conditions following PDT are critical for effective tumor treatment (Tang et al., 2023; Zhang et al., 2020). Fortunately, hypoxia-activated prodrugs have emerged as innovative therapeutics for specific elimination of these hypoxic cells (Baran and Konopleva, 2017; Ma et al., 2021; Yang et al., 2022a; Wang et al., 2022). While exhibiting no cytotoxicity in normoxic conditions, tirapazamine (TPZ) can be reduced into cytotoxic radicals that responsible for killing cells specifically under hypoxic conditions (Zhang et al., 2022). Consequently, the de-livery of photosensitizers and hypoxia-activated prodrugs by nanocarriers is promising to realize more effective PDT and chemotherapy for cancer treatment.

Most of photosensitizers and hypoxia-activated prodrugs are hydrophobic and frequently interact non-specifically with normal tissues and cells, leading to inadequate accumulation in tumors and significant systemic toxicity *in vivo* (Xiong et al., 2024; Du et al., 2020; Song et al., 2021; Zhou M. et al., 2020; Ma et al., 2020). Recently, the utilization of nanocarriers has represented a significant advancement in drug delivery systems, enhancing the solubility, circulation time in the bloodstream, and biodistribution of these low molecular weight agents (Zhou et al., 2021; Zhou S. et al., 2020; Yang et al., 2022b). Polyphosphoester (PPE), characterized by its repetitive phosphoester linkages, is a biodegradable polymer that can be easily functionalized with various side groups (Zhang et al., 2015; Pei et al., 2019). Recent studies have demonstrated its potential biocompatibility, garnering significant interest in the fields of biomaterials and tissue engineering. PPE can be rationally designed to self-assemble into micelles, vesicles, or hydrogels for the encapsulation of various therapeutic agents, enabling controlled drug release (Hu et al., 2015; Wang et al., 2009; Zhang et al., 2018). Through modification by different moieties, the degradation pattern of PPE could be tailored to achieve more precise drug delivery while providing drug protection and treatment for malignant cancers (Yilmaz and Jérôme, 2016; Li W. et al., 2023). Moreover, PPE could be used as the surface modification of nanocarriers, serving as an alternative to traditional polyethylene glycol, to facilitate the cargoes retention in the bloodstream (Simon et al., 2018; Li et al., 2018). In addition, the ability of PPE to degrade into non-toxic byproducts enhances their appeal in tissue engineering and regenerative materials (Riva et al., 2019). Wang’s group and other researchers have developed diverse nanocarriers that utilize polyphosphoesters for effective chemotherapy, photothermal therapy, and photodynamic therapy (Sun et al., 2015; Zhang et al., 2019; Ma and Sun, 2020; Li et al., 2017; Li X. et al., 2023). To the best of our knowledge, there have been no reports of polyphosphoester-based nanocarriers that co-deliver both photosensitizers and hypoxia-activated prodrugs.

**FIGURE 1:**
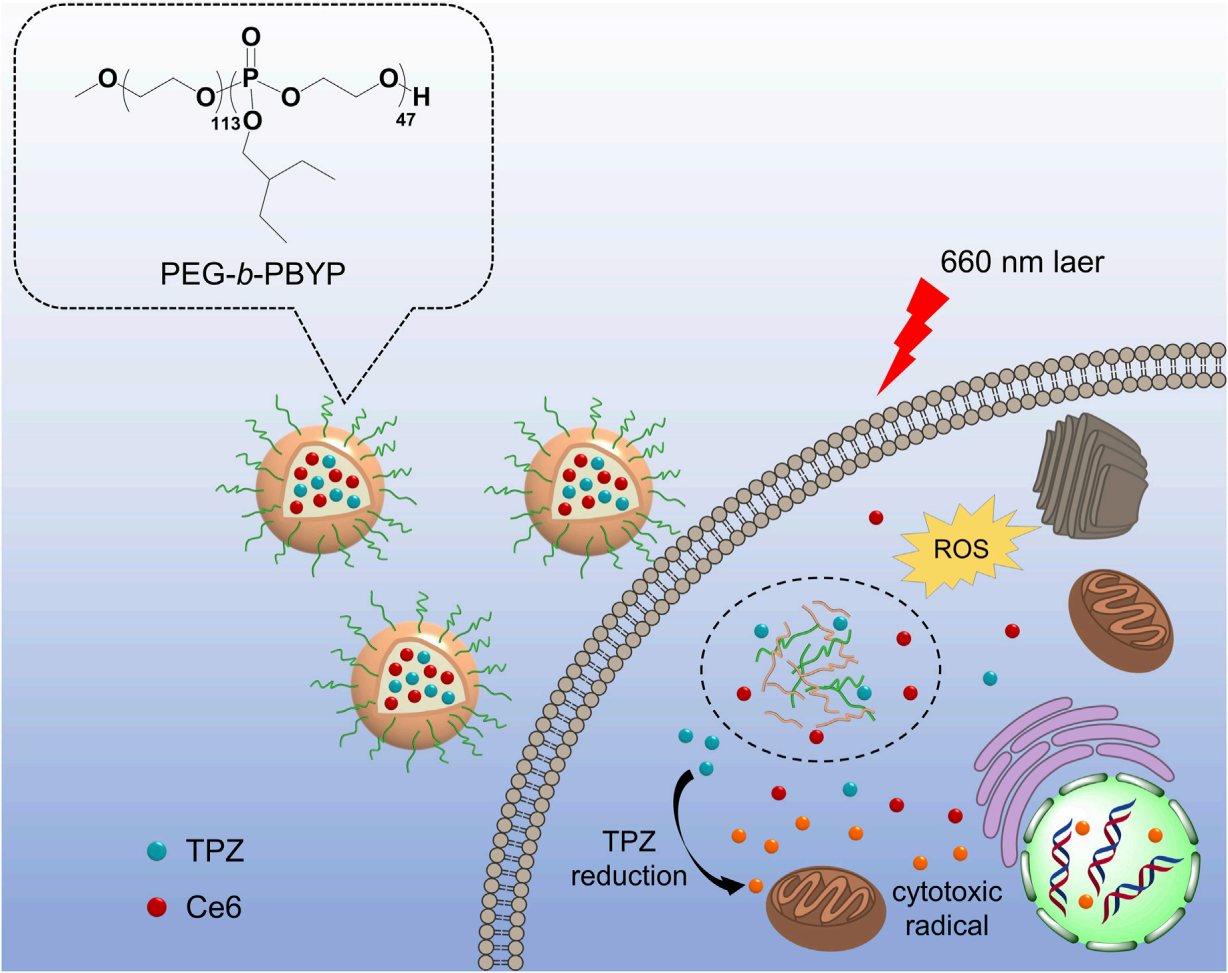
Schematic illustration of NP_CT_ for combined PDT and TPZ-assisted chemotherapy against 4T1 breast cancer. After the effective PDT for normoxic cells, the TPZ was activated by hypoxic conditions, allowing for targeted chemotherapy to remaining hypoxic cells.

In this study, we developed an amphiphilic PEGylated polyphosphoester (PEG-*b*-PBYP) by incorporating a hydrophobic moiety to facilitate the efficient loading of chlorin e6 (Ce6, a photosensitizer) and TPZ (a hypoxia-activated prodrug), aimed at enhancing the efficiency of traditional PDT (Figure 1). As illustrated in Figure 1, both cargoes were effectively encapsulated within nanosized micelles (NP_CT_) that self-assembled from PEG-*b*-PBYP through hydrophobic-hydrophobic interactions. Following intravenous (*i.v.*) injection, PEGylation on N_PCT_ protects the cargoes from rapid clearance from the bloodstream and aids their navigation to tumor sites via passive accumulation mechanisms. Once the nanocarriers are internalized by tumor cells, 660 nm laser irradiation in conjunction with Ce6 initiates the PDT process, resulting in ROS-induced cancer cell death. Simultaneously, the PDT process depletes surrounding oxygen, exacerbating the hypoxic microenvironment. Subsequently, the released TPZ is selectively converted into SR 4317 and cytotoxic radicals by NADPH-dependent reductase, effectively killing the residual cells after PDT through hypoxia-activated chemotherapy. The combined efficacy of NP_CT_ and 660 nm laser radiation was rigorously assessed both *in vitro* and *in vivo*.

## Materials and methods

### Materials and animals

2-Ethylbutoxy-2-oxo-1, 3, 2-dioxaphospholane (BYP) and PEGylated PBYP (PEG-*b*-PBYP) were synthesized via previous report by our group (Zhang et al., 2019). Ce6 and TPZ were obtained from Beijing J&K Scientific Co., Ltd. (China). Cell counting kit-8 (CCK-8) was purchased from Beyotime Biotech Inc. (Shanghai, China). Dulbecco’s modified Eagle’s medium (DMEM) and fetal bovine serum (FBS) were purchased from Life Technologies Corporation (Gibco, United States). Other chemicals, unless otherwise specified, were of analytical grade and used as received.

BALB/c mice (female, 6 weeks old) were purchased from Beijing HFK Bioscience Co., Ltd. (China). All animals received care in compliance with the guidelines outlined in the Guide for the Care and Use of Laboratory Animals and all procedures were approved by the Tianjin Medical University Animal Care and Use Committee.

### Preparation and characterization of NP_CT_

The micelles (NP_CT_) encapsulated Ce6 and TPZ were fabricated using a nanoprecipitation strategy. In brief, 20.0 mg of PEG-b-PBYP, 2.0 mg of Ce6, and 2.0 mg of TPZ were dissolved in 2.0 mL of DMSO. The mixture was then added dropwise to 20.0 mL of ddH_2_O while stirring gently. After the stirring for 30 min, DMSO, along with any unencapsulated Ce6 and TPZ, was removed via dialysis (molecular weight cut-off of 3,400) against ddH_2_O at 4°C, followed by centrifugation at 1,000 *g*. Similarly, micelles loaded with Ce6 or TPZ were prepared using the same method.

### Drug release from Ce6&TPZ encapsulated micelles

To assess the *in vitro* release of TPZ, suspensions of NP_C_, NP_T_, and NP_CT_ (with a Ce6 content of 300 μg) were placed in dialysis tubes with a molecular weight cut-off of 3,500 and immersed in phosphate buffer at pH 7.4 and kept in the dark. The dialysis tubes were gently shaken at 37°C at 60 rpm. At certain time intervals, the outside incubation solution was collected and replaced with fresh buffer. The TPZ content in collected solution was quantified using HPLC.

### Cellular uptake and PDT outcome of NP_CT_

4T1 cells were plated on glass coverslips at a density of 1 × 10^5^ and incubated for 12 h. Then, the cells were treated with NP_CT_ for 6 h at 37°C. After incubation, the cells were rinsed three times with PBS, fixed using 4% paraformaldehyde for 15 min and stained with DAPI to visualize the nuclei. Finally, the coverslips were mounted onto glass slides to observe the cellular uptake by confocal microscopy (CLSM).

4T1 cells were cultured in 12-well plates at a density of 2 × 10^5^ and incubated for 12 h. The cells were then incubated with NP_C_ or NP_CT_ for 2, 4, or 6 h. Following incubation, the cells were rinsed with cold PBS and collected. The intracellular Ce6 content was determined using a fluorescence spectrophotometer after cell lysis.

### Cytotoxicity assay of NP_CT_

4T1 cells were plated in a 96-well plate at a density of 5,000 cells per well and incubated with NP_C_, NP_T_, or NP_CT_. Following 12 h of incubation, the medium was replaced with fresh DMEM, and the cells were subjected to 660 nm laser (0.5 W/cm^2^) for 10 min. After an additional 60 h of incubation under normoxic or hypoxic (1% O_2_) condition, cell viability was assessed using a standard CCK-8 kit, with measurements taken using a Bio-Rad 680 microplate reader.

### *In vivo* pharmacokinetical profile and tumor accumulation of NP_CT_

BALB/c mice were received intravenous injections of free Ce6, NP_C_, or NP_CT_ with a concentration of Ce6 at 10 μg per gram body weight (n = 4). Plasma samples (100 μL) were collected from the retroorbital plexus of the mice at various time points: 0.08, 0.5, 1, 2, 4, 8, 12, 24, 48, and 72 h after injection. The plasma concentration of Ce6 was subsequently analyzed using an above-mentioned method.

Furthermore, BALB/c mice bearing 4T1 xenografts also received systemic injections of Ce6, NP_C_, or NP_CT_ at the same dosage (n = 4). At 12 and 24 h post injection, the mice were sacrificed, and solid tumors were collected. The quantitative distribution of Ce6 in tumor tissues was determined through HPLC.

### Anticancer treatment

BALB/c mice bearing 4T1 xenografts were randomly assigned to five groups (n = 5). When the tumor grew to approximately 50 mm^3^, mice were received intravenous injections of PBS, free Ce6+TPZ, NP_C_, NP_T_, or NP_CT_, with a TPZ concentration of 5 μg per gram of body weight on days 0, 7, and 14. Following a 24-hour period post-injection, the tumor regions were irradiated with 660 nm laser for 10 min at a power density of 0.5 W/cm^2^. Tumor volume and body weight were tracked every 3 days, with tumor volume calculated using the formula: tumor volume = 0.5 × length × width^2^. After the final measurement, the primary tumors and major organs were harvested and fixed in 4% paraformaldehyde for hematoxylin and eosin (H&E) staining and subsequent histopathological analysis.

## Results

### Preparation and characterization of NP_CT_

**FIGURE 2:**
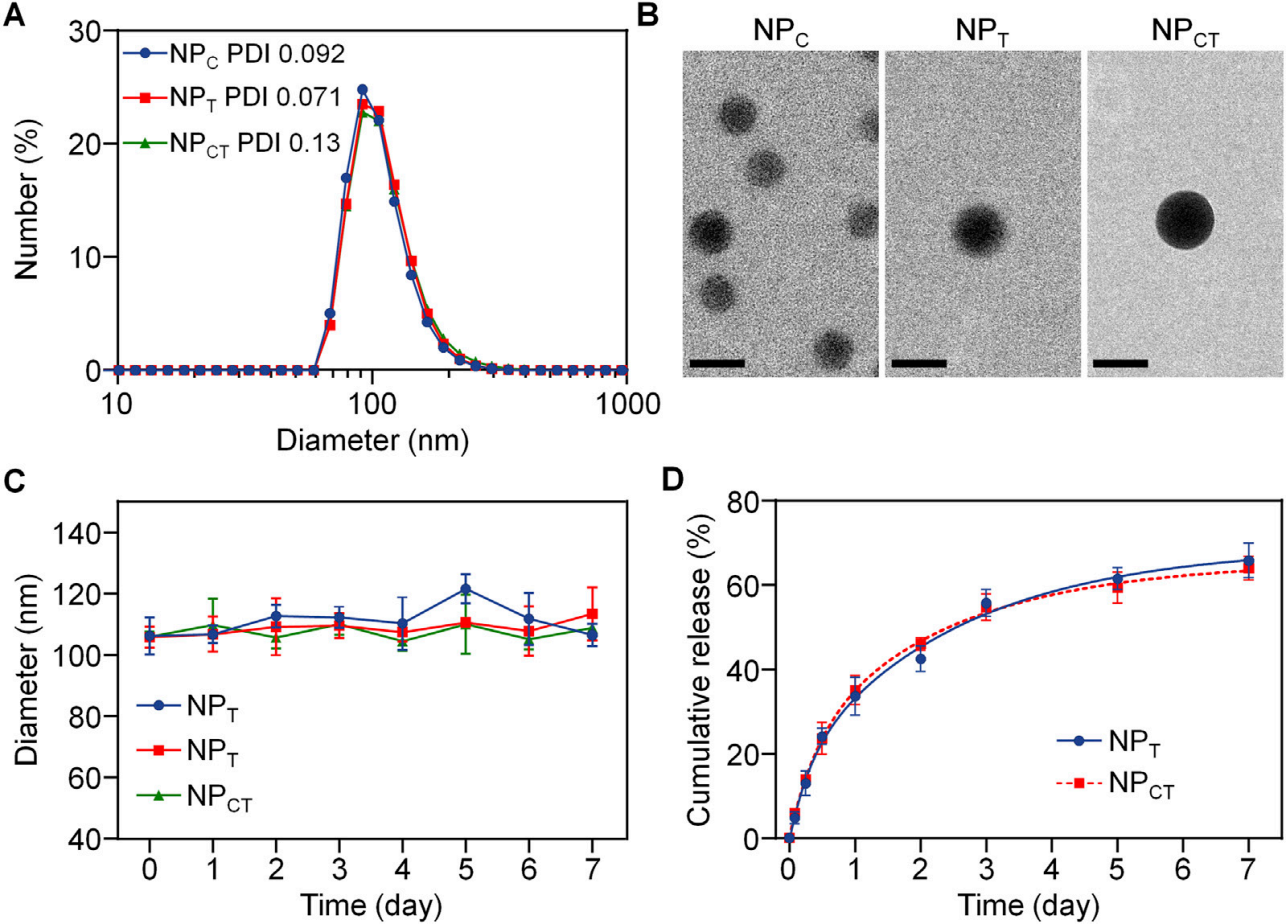
**(A)** Hydrodynamic diameter of NP_C_, NP_T_ and NP_CT_ measured by DLS. **(B)** TEM images of NP_C_, NP_T_ and NP_CT_. The scale bar is 100 nm. **(C)** Diameter change of NP_C_, NP_T_ and NP_CT_ following incubation for 168 h. **(D)** Release pattern of TPZ from NP_T_ and NP_CT_ for 7 days.

We employed a nanoprecipitation route to incorporate Ce6, a photosensitizer, along with TPZ, a hypoxia activated prodrug, into the hydrophobic core, resulting in nanoparticles referred to as NP_CT_. Additionally, micelles encapsulating either Ce6 (denoted by NP_C_) or TPZ (denoted by NP_T_) were fabricated using a similar approach. As illustrated in Figure 2A, dynamic light scattering (DLS) measurements indicated that the average diameter of NP_C_, NP_T_, and NP_CT_ was around 110 nm. Transmission electron microscopy (TEM) images (Figure 2B) revealed that three nanoparticulate formulations exhibited a typica micellar structure with compact and spherical cores, also approximately 100 nm in diameter. The zeta potential of NP_C_, NP_T_, and NP_CT_ was −20.8, −23.6, and −21.0 mV, respectively. The drug loading content (DLC) and encapsulation efficiency (EE) for Ce6 and TPZ in NP_CT_ was shown in Supplementary Table S1, and the DLC of Ce6 and TPZ was determined to be 2.59% and 2.17% by UV-vis spectrophotometer, which was close to that of NP_C_ and NP_T_, respectively. Furthermore, after incubating in DMEM with 10% FBS at 37°C for 168 h, no significant alterations in diameter of NP_CT_ were found (Figure 2C), indicating protective PEGylation and advanced stability in biological environments. We then investigated the release pattern of TPZ from NP_T_, and NP_CT_ at pH 7.4. As illustrated in Figure 2D, under neutral conditions, more than 60% of the total TPZ was released from either NP_T_, or NP_CT_ after 168 h, which may be attributed to the degradation of polyphosphoesters.

### Cellular uptake and intracellular PDT effect of NP_CT_ *in vitro*

**FIGURE 3:**
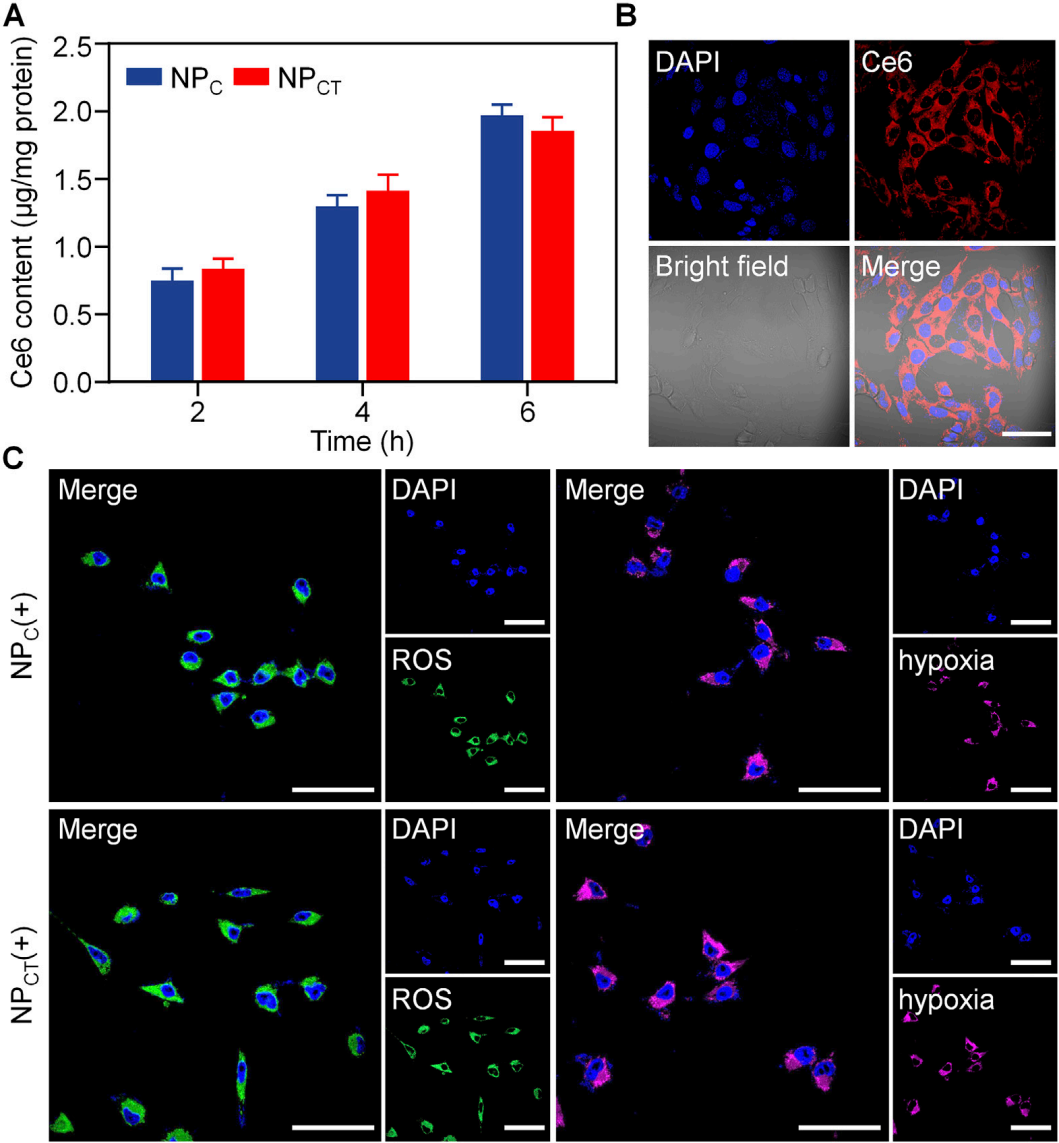
**(A)** Internalized Ce6 content after incubating 4T1 cells with NP_C_ and NP_CT_ for 2, 4, or 6 h. **(B)** Confocal observation of cellular uptake and subcellular distribution of Ce6 fluorescence after incubation with NP_CT_ for 6 h. The scale bar is 50 μm. **(C)** Confocal images of 4T1 cells exposed with 660 nm laser irradiation. The scale bar is 50 μm. (+): 660 nm laser irradiation.

We subsequently investigated the intracellular concentration of Ce6 through a quantitative approach. 4T1 cells were treated with free curcumin, NP_C_, or NP_CT_ for durations of 2, 4, or 6 h, and the Ce6 uptake was analyzed using fluorescence spectrophotometry. As depicted in Figure 3A, an increase in incubation time corresponded with a gradual rise in intracellular Ce6 levels for 4T1 cells exposed to either NP_C_ or NP_CT_. Following a 6-hour incubation, NP_CT_ enabled the uptake of 1.86 μg of Ce6 per mg of protein in 4T1 cells. Additionally, the internalization of NP_CT_ by 4T1 cells was validated using CLSM. As displayed in Figure 3B, substantial Ce6 fluorescence was found in the cytoplasm after incubating NP_CT_ with 4T1 murine breast cancer cells for 6 h, suggesting its effective cellular uptake. Next, NP_CT_-induced ROS generation and hypoxic conditions was studied by CLSM using specific fluorescent probes. Upon 660 nm laser irradiation, both NP_C_ and NP_CT_ displayed the strong ROS signal intracellularly, whereas the local hypoxic microenvironment was formed comparably during the PDT process (Figure 3C).

### Toxicity effect of NP_CT_ *in vitro*

**FIGURE 4:**
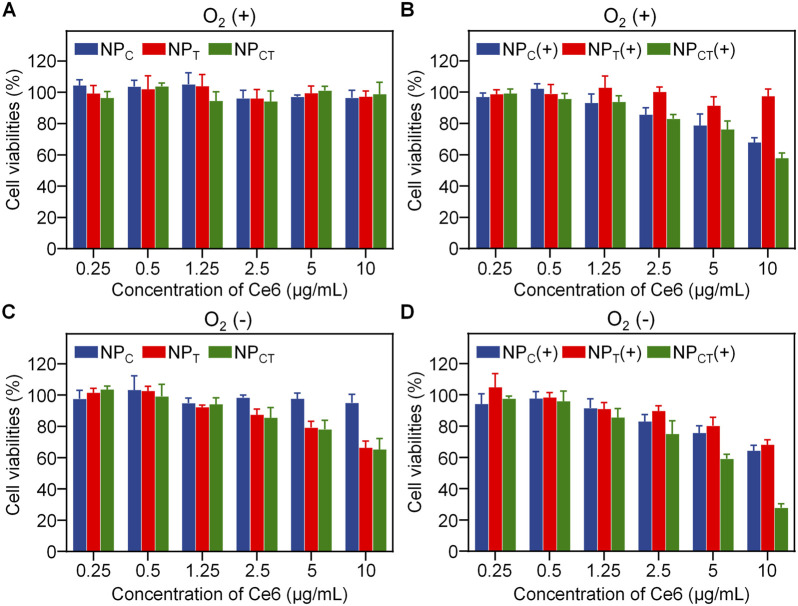
Cytotoxicity of 4T1 cancer cells treated with NP_C_, NP_T_, or NP_CT_ under normoxic **(A, B)** or hypoxic **(C, D)** condition. (+): 660 nm laser irradiation.

The cytotoxicity assay of NP_C_, NP_T_, or NP_CT_ on 4T1 cell line was conducted using the CCK-8 kit. It was found that all nanocarriers displayed no significant cytotoxic effects under normoxic condition, even at Ce6 concentration reaching 10 μg/mL (Figure 4A). After the cargoes encapsulation, NP_CT_ acquired the ability for photosensitization reaction and hypoxia-activated chemotherapy. Therefore, we further evaluated its cancer cell-killing efficacy against 4T1 cells when exposed to 660 nm laser irradiation with sufficient O_2_ supply. As shown in Figure 4B, NP_C_ and NP_CT_ treatments produced considerable anticancer effects with the application of a 660-nm laser, with cell viability decreasing to approximately 67.6% ± 3.1% [NP_C_(+)] and 57.5% ± 3.4% [NP_CT_(+), (Ce6) = 10 μg/mL], respectively. Although the PDT process consumed O_2_ oxygen, TPZ was not activated under normoxic condition, thereby resulting in the negligible cytotoxicity of NP_T_. Similarly, NP_T_ and NP_CT_ were evaluated as chemotherapeutic agents under hypoxic condition, yielding comparable findings. As shown in Figure 4C, TPZ-loaded micelles reduced cell viability to about 66.3% ± 4.2% (NP_T_) and 65.1% ± 7.1% (NP_CT_) at the maximum concentration. To further explore the therapeutic outcomes of combined PDT and hypoxia-activated chemotherapy, 4T1 cells were incubated with NP_CT_ and subsequently exposed to 660 nm laser and incubation for 60 h under hypoxic condition in sequence. As expected, the combined treatment [NP_CT_(+)] notably decreased cell viability to 27.7% ± 2.6% at the highest Ce6 concentration (10 μg/mL, Figure 4D). By contrast, NP_C_(+) treatment under hypoxia led to only a moderate reduction in cell growth (64.3% ± 3.4%), which comparable to that of NP_C_(+) under normoxic condition, indicating that the combination therapy of NP_CT_ is more effective *in vitro*.

### Pharmacokinetic and biodistribution of NP_CT_ *in vivo*

**FIGURE 5:**
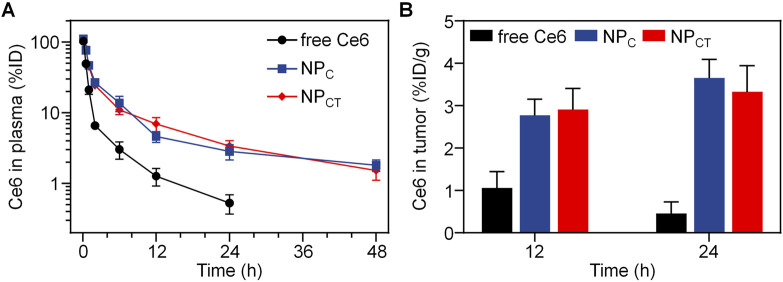
**(A)** Plasma Ce6 content versus time after intravenous injection of free Ce6, NP_C_ or NP_CT_ (n = 4, mean ± SD). **(B)** Quantitative Ce6 concentration of different formulations in tumor tissues at 12 and 24 h post-injection.

The pharmacokinetics of free Ce6, NP_C_ and NP_CT_ were assessed using female BALB/c mice without tumors. At 0.08, 0.5, 1, 2, 6, 12, 24 and 48 h post-injection, the blood samples of mice were collected to measure the Ce6 concentration in the plasma. As illustrated in Figure 5A, both NP_C_ and NP_CT_ demonstrated extended retention in the bloodstream, whereas free Ce6 was quickly cleared following *i.v.* injection. At 24 h post-injection, plasma concentration of Ce6 was detected at 0.53% ± 0.16% of the injected dose for free Ce6-treated mice. In contrast, both NP_C_ and NP_CT_ prolonged the Ce6 circulation in bloodstream to 1.82% ± 0.34% and 1.53% ± 0.42% of injected dose even at 48 h post-injection, respectively. Furthermore, we systemically injected free Ce6, NP_C_, or NP_CT_ into 4T1 tumor-bearing BALB/c mice, and the tumor tissues were collected at 12 and 24 h post-injection to quantify the Ce6 content. As shown in Figure 5B, there was only 0.46% ± 0.28% of injected dose per gram tumor of Ce6 was detected in free Ce6 group at 24 h post-injection. In contrast, the tumor accumulation of both NP_C_ and NP_CT_ was 3.67% ± 0.44% and 3.39% ± 0.63% of injected dose per gram tumor at the same time interval, which was 7.98- and 7.37-fold greater than that of free Ce6 group, respectively.

### Tumor growth inhibition *in vivo*

**FIGURE 6:**
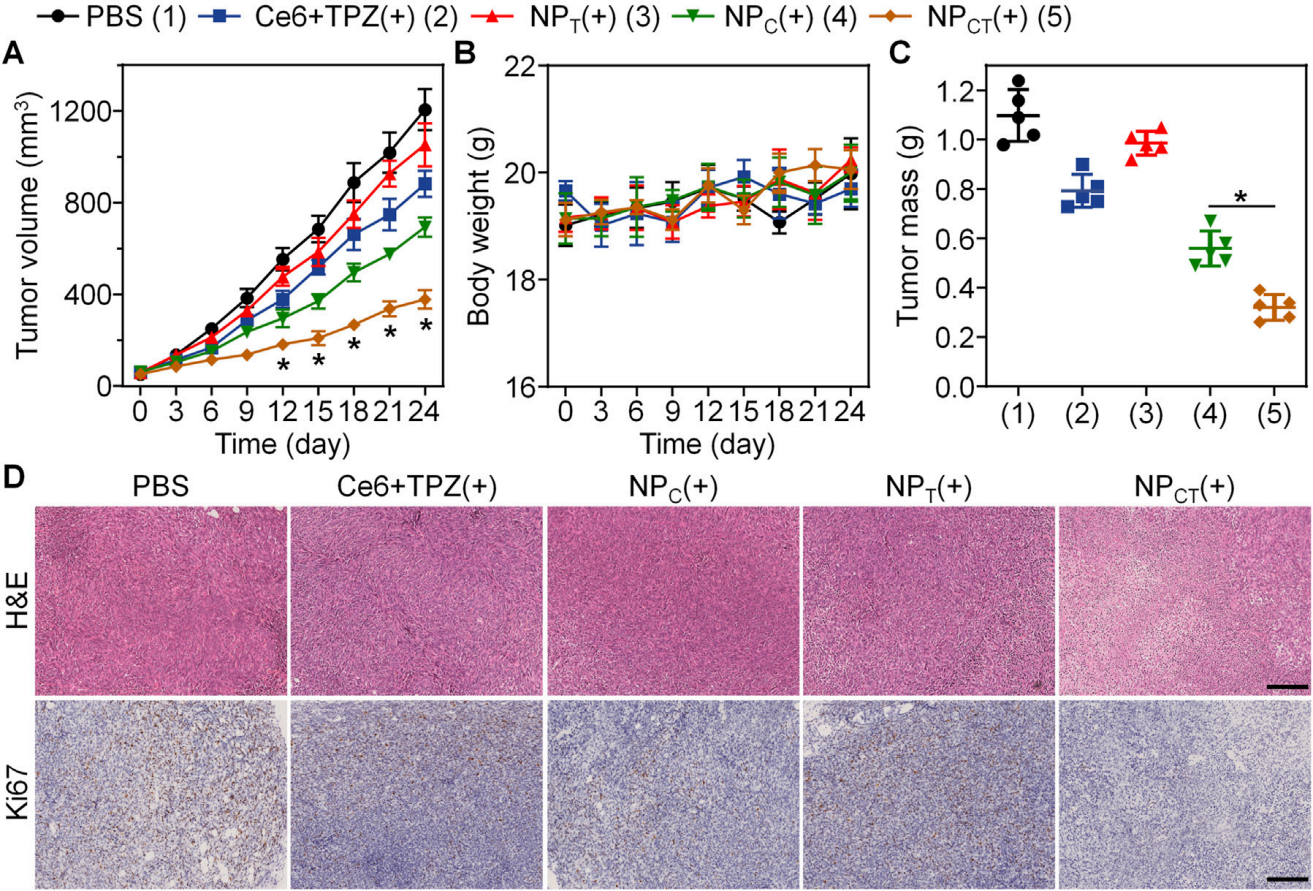
Combined PDT and hypoxia-activated chemotherapy against 4T1 tumor on animal level. **(A)** Tumor growth in 4T1 tumor-bearing mice receiving different treatments. *p < 0.05 vs*.* NP_C_(+). **(B)** Body weight changes in BALB/c mice during the therapeutical period. **(C)** Tumor weight on day 24 of the therapeutical period. *p < 0.05. **(D)** H&E and Ki67 staining of tumor sections from various groups. The scale bar is 200 μm.

5 × 10^5^ 4T1 cells were implanted into BALB/c mice, which were then randomly assigned to one of five treatment groups when the tumor volume grew to about 50 mm^3^. The mice in five groups received PBS, Ce6+TPZ, NP_C_, NP_T_, or NP_CT_ with an equivalent dose of 5 mg/kg body weight of TPZ administered through systemic injection every 7 day for three times, and the last four groups exposed to 660 nm laser at 24 h post-injection. During the whole treatment period, we monitored tumor size and body weight every 3 day. Because of both rapid clearance and insufficient tumor accumulation, there was no significant difference regarding tumor growth between PBS and Ce6+TPZ(+) group (Figure 6A). In comparison to the negative PBS control group, where average tumor size exceeded 1,200 mm^3^ by day 24, treatment with NP_C_(+) resulted in a partial reduction of tumor growth to 693.96 ± 41.87 mm^3^. Notably, the NP_CT_(+) group demonstrated the highest level of tumor growth inhibition, achieving a tumor inhibition rate (TIR) of 68.55%. As shown in Figure 6B, Throughout the 24 days of monitoring, body weight remained relative stable among all groups, suggesting good tolerance of NP_CT_(+) treatment. Upon completion of the treatment, tumor tissues were removed and weighed. The lowest tumor weight was observed in the NP_CT_(+) group, further verifying its potent antitumor activity (Figure 6C). Histopathological evaluation of the tumor tissue through H&E staining demonstrated a downregulation of Ki67, a marker of cell proliferation, in the NP_CT_(+) group (Figure 6D).

### Biosafety evaluation *in vivo*

**FIGURE 7:**
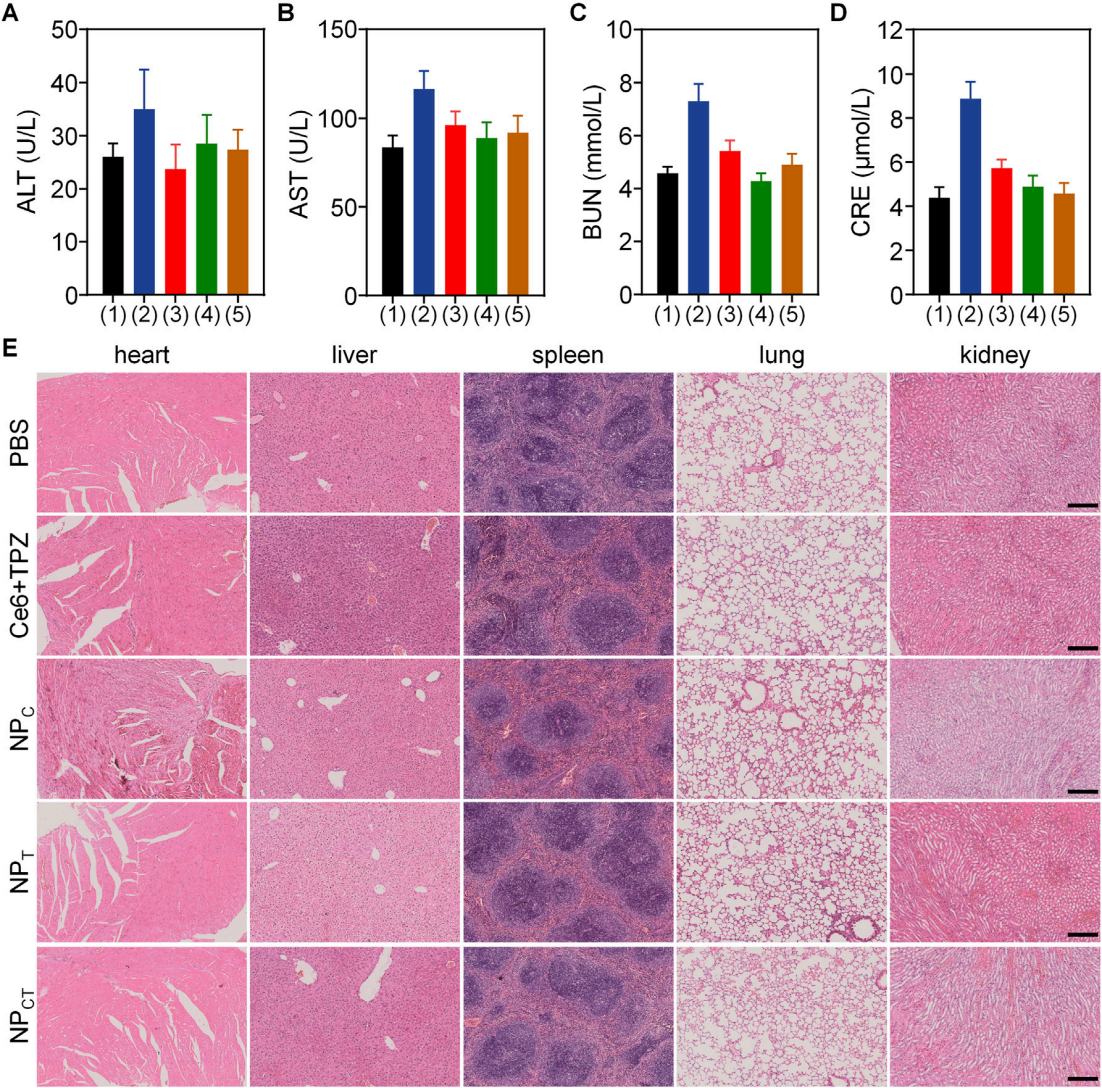
Biocompatibility assay of PBS, free drug and different nanocarriers. **(A–D)** Content of ALT, AST, BUN, and CRE in BALB/c mice received *i.v.* injections with PBS (1), Ce6+TPZ (2), NP_C_ (3), NP_T_ (4), or NP_CT_ (5). **(E)** Histopathological H&E analyses of major organs form mice following different treatments. The scale bar is 200 μm.

To evaluate the *in vivo* biosafety of the designed NP_CT_, we intravenously administered PBS, free Ce6+TPZ, NP_C_, NP_T_, or NP_CT_ to female BALB/c mice via tail vein over 7 days while monitoring their body weight. As depicted in Supplementary Figure S1, the mice receiving nanoparticulate carriers displayed relatively unchanging body weights, suggesting minimal systemic toxicity associated with NP administration. Subsequently, we collected plasma samples from the retro-orbital plexus to analyze the routine blood indices and biochemical indicators by ELISA assays. In contrast to the free Ce6+TPZ injection, there were no notable differences in routine blood, alanine aminotransferase (ALT), aspartate aminotransferase (AST), blood urea nitrogen (BUN), and creatinine (CRE) between the PBS negative control group and the nanoparticulate vehicles (Figures 7A–D; Supplementary Figure S2). Histopathological H&E staining of the heart, liver, spleen, lung, and kidney (Figure 7E) further indicate the expected biosafety of the proposed nanocarriers based on polyphosphoesters and the decreased harmfulness following the loading of both agents.

## Discussion

The amphiphilic nature of PEG-*b*-PBYP, as demonstrated by Wooley et al., allows for the encapsulation of hydrophobic agent in the presence of hydrophobic PBYP segment (Zhang et al., 2015). Our results indicate that all nanoparticles successfully encapsulated Ce6 and/or TPZ through hydrophobic-hydrophobic interactions and remained stable n buffer for 1 week due to their PEGylated surface. Furthermore, TPZ can be efficiently liberated from the core of the nanocarriers, suggesting the potential for subsequent hypoxia-activated chemotherapy following the PDT process. These data confirmed that both Ce6 and TPZ could be successfully encapsulated by PEGylated polyphosphoesters, and TPZ could be efficiently released to function as a chemotherapeutic agent.

Considering the mechanisms of action and cellular uptake sites, the cellular uptake of both Ce6 and TPZ is essential for their roles as photosensitizer and chemotherapeutic agent, respectively. We studied the cellular uptake of NP_CT_ through both quantitative analysis and confocal microscopy. Following irradiation with a 660 nm laser, considerable production of ROS and the presence of residual hypoxic conditions were confirmed using different fluorescent probes. The results indicated that NP_CT_ could exacerbate the intracellular hypoxia to potentially facilitate TPZ activation for chemotherapy.

Previous studies have indicated that PEGylated polyphosphoesters are promising candidates for drug delivery, primarily because of their favorable biodegradability and biocompatibility (Li et al., 2024; Chen et al., 2016). In the absence of drug loading, NP exhibited negligible cytotoxicity towards 4T1 cells even with insufficient O_2_ supply. Numerous studies, however, have confirmed that robust PDT can exacerbate hypoxia levels due to oxygen consumption. As expected, NP_CT_ killed more breast cancer cells under hypoxic conditions than NP_C_ group. According to our design, the PEG on the NP_CT_ surface prevents interactions with macrophages in the bloodstream, thereby prolonging the cargo circulation. In contrast to free drug, which was rapidly cleared from the bloodstream, NP_CT_ realized prolonged retention of cargoes, and the significantly prolonged circulation of encapsulated photosensitizer could be primarily attributed to the PEGylation on the NP surface. On the other hand, NP_CT_ also facilitated the accumulation of Ce6 in tumor lesions at both 12 and 24 h post-injection. As previously discussed, the promoted Ce6 distribution in tumor tissues of nanosized NP_C_ and NP_CT_ could be linked to both extended blood circulation and the enhanced permeability and retention (EPR) effect.

The extended retention of both the photosensitizer and the hypoxia-activated drug in the bloodstream, along with enhanced accumulation in targeted lesions, motivated us to investigate the therapeutic outcome of NP_CT_ against the murine 4T1 breast cancer *in vivo*. We monitored the 4T1 tumor growth throughout the entire therapeutic window and measured the tumor mass after treatment. Both assessments demonstrated the most remarkable tumor growth inhibition in the NP_CT_(+) group. In this study, nanocarriers were self-assembled from PEGylated polyphosphoesters that can be degraded by phosphoesterases *in vivo*, ensuring favorable biocompatibility. The assessments of live and kidney function and pathological analyses of major organs further support our hypothesis. Collectively, these findings confirmed that NP_CT_(+) significantly inhibited tumor growth through the combination of PDT and hypoxia-activated chemotherapy with satisfactory biosafety *in vivo*.

## Conclusion

In summary, we developed a PEGylated polyphosphoester-based nanocarrier designed for the simultaneous delivery of Ce6 and TPZ to facilitate a combination of PDT and hypoxia-activated chemotherapy. All results indicated that NP_CT_ serves effectively as both a photosensitizer and a chemotherapeutic agent against malignant triple negative breast cancer. The combination approach of PDT and hypoxia-activated chemotherapy resulted in significantly enhanced anticancer effects compared to the application of either therapy alone, as evidenced by promoted cancer cell death and 4T1 tumor growth inhibition in both *in vitro* and *in vivo* studies. Notably, 660 nm laser irradiation alone did not induce any obvious cell death, confirming the biosafety of the light at the power density applied on cellular level. Furthermore, the prepared NP_CT_ displayed advantageous biocompatibility and exhibited no evident toxicity at concentrations necessary for cancer treatment. This work not only highlights the potential of polyphosphoesters-based nanocarriers for effective combination therapy but also broadens their application in future, paving the way for the exploration of polyphosphoesters in various therapeutical strategy, which may significantly contribute to their clinical application.

## Data Availability

The original contributions presented in the study are included in the article/Supplementary Material, further inquiries can be directed to the corresponding author.
