# Supplementary material for: Biocompatible nanoparticles self-assembled by PEGylated polyphosphoesters for combination of photodynamic therapy and hypoxia-activated chemotherapy against breast cancer
Source: Front Pharmacol. 2024 Dec 23;15:1529631. doi: 10.3389/fphar.2024.1529631 (PMC11701217; doi:10.3389/fphar.2024.1529631)
Supplement: Supplementary file 1 [file DataSheet1.docx]

Supplementary Material

**Characterization Method**

The size and zeta potential measurements were carried out in aqueous solution using a Malvern ZS90 dynamic light scattering instrument with a He-Ne laser (633 nm) and 90° collecting optics. The data were analyzed using Malvern Dispersion Technology Software 5.10. Transmission electron microscope (TEM) measurements were performed on a JEOL 2010 high-resolution transmission electron microscope with an accelerating voltage of 200 kV.

**Biosafety Evaluation**

Female BALB/c mice received i.v. injection of PBS, free Ce6+TPZ, NP_C_, NP_T_, or NP_CT_ over seven days. On day 8, mice were euthanized and blood samples were obtained through cardiac puncture for biochemical analysis. The collected blood was centrifuged at 3000 rpm for 10 minutes to separate the serum. Serum levels of alanine aminotransferase (ALT), aspartate aminotransferase (AST), blood urea nitrogen (BUN), and creatinine (CRE) were assessed using an automated biochemical analyzer to evaluate liver and kidney function. Additionally, the heart, liver, spleen, lungs, and kidneys from each mouse were excised for histopathological examination. The organs were fixed in 4% paraformaldehyde for 24 hours, embedded in paraffin, sliced into 6 μm sections, and stained with hematoxylin and eosin (H&E).

**Statistical Analysis**

Statistical significance was analyzed using an independent samples t-test, and the differences were considered significant for *p < 0.05. Unless specially noted, data are shown as mean ± SD.


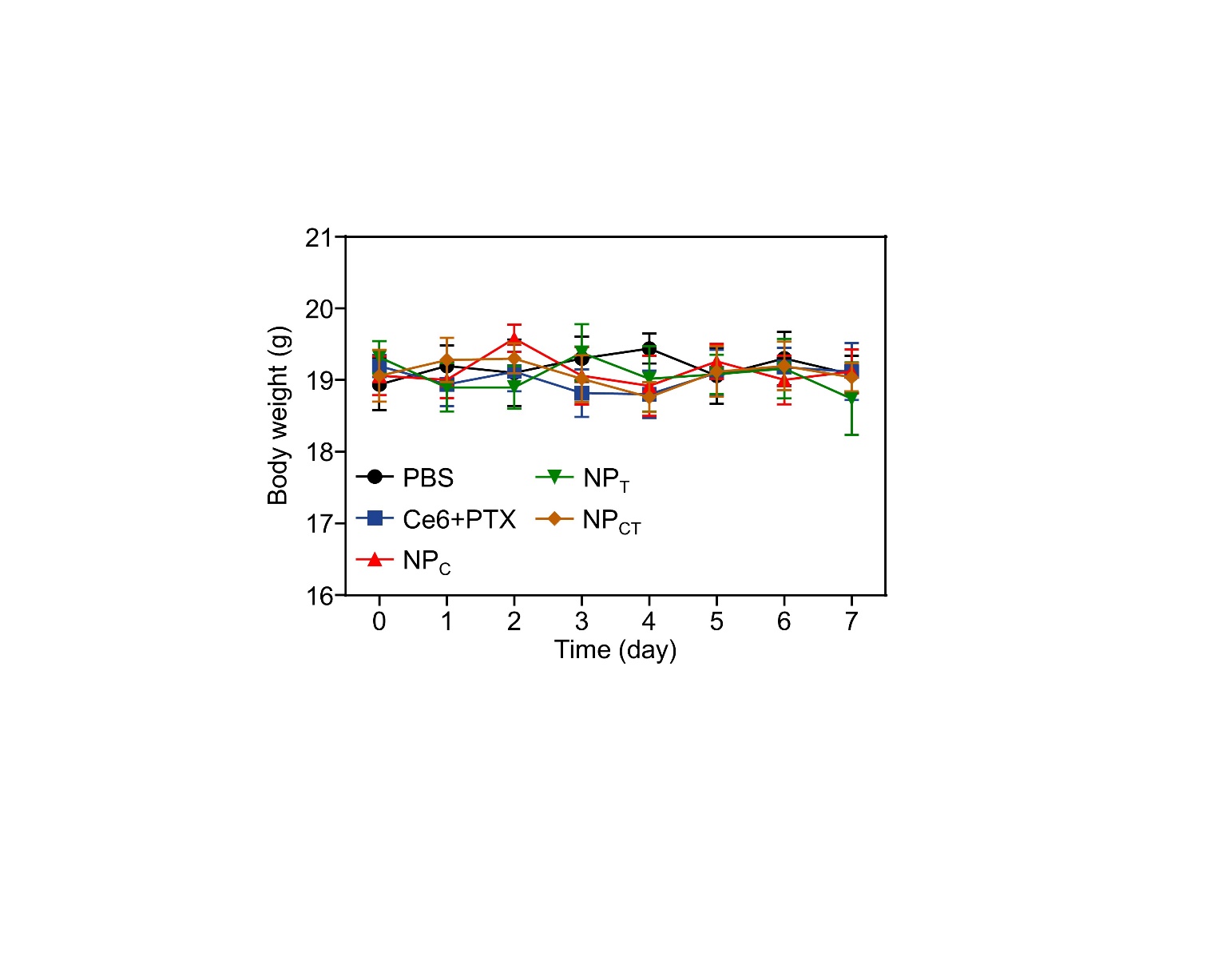


**Figure S1*.*** Body weight of mice after different treatments.


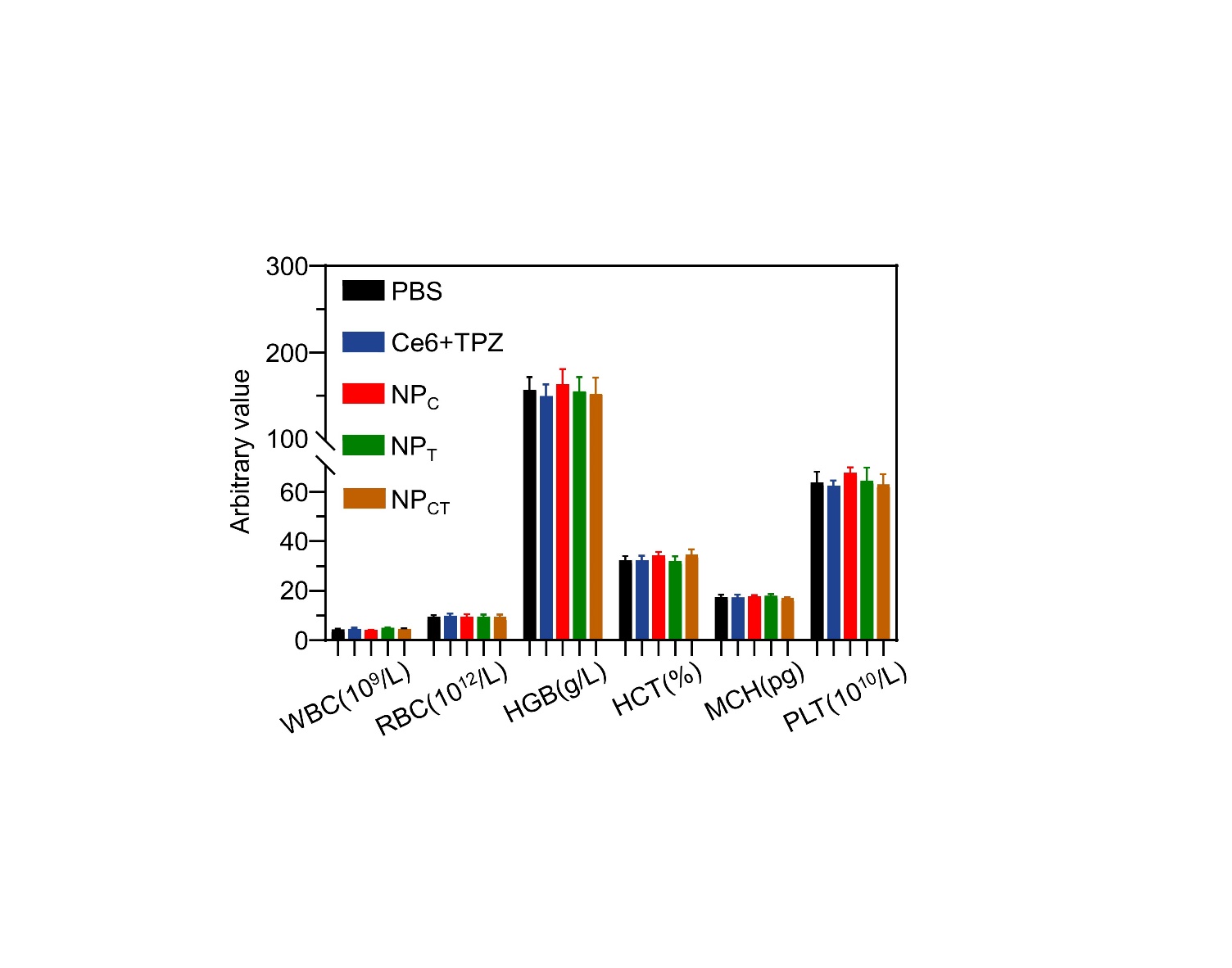


**Figure S2*.*** Hematology analysis of the mice after different treatments.

**Table S1.** Drug loading content (DLC) and encapsulation efficiency (EE) of Ce6 and TPZ for NP_C_, NP_T_, and NP_CT_.

|  | DLC (%) | | EE (%) | |
| --- | --- | --- | --- | --- |
|  | Ce6 | TPZ | Ce6 | TPZ |
| NP_C_ | 2.98 |  | 28.76 |  |
| NP_T_ |  | 2.63 |  | 23.14 |
| NP_CT_ | 2.59 | 2.17 | 21.83 | 19.42 |
